# Fecal Microbiota Dysbiosis and Diarrhea-Associated Bacterial Taxa in Post-Weaning Dairy Calves

**DOI:** 10.3390/microorganisms14102171

**Published:** 2026-09-25

**Authors:** Jian Zhang, Jianshen Zhu, Yunxia Li, Xiaoli Hao, Zhimin Dong, Zhiguo Gao, Xihe Li

**Affiliations:** 1 College of Agriculture, Animal Husbandry and Food Science, Hetao College, Bayannur 015000, China; wo-de-aizj@163.com (J.Z.);; 2 School of Materials Science and Engineering, Shanghai Jiao Tong University, Shanghai 200240, China; 3 Inner Mongolia Saikexing Institute of Breeding and Reproductive Biotechnology in Domestic Animal, Hohhot 011517, China; 4 National Center of Technology Innovation for Dairy Co., Ltd., Hohhot 011517, China

**Keywords:** post-weaning calf, diarrhea, fecal microbiota, 16S rRNA sequencing, microbial dysbiosis

## Abstract

Post-weaning diarrhea is a prevalent gastrointestinal disorder in intensive dairy calf production, causing impaired growth, elevated mortality and widespread excessive antimicrobial use. While fecal microbiota dysbiosis is well implicated in diarrhea pathogenesis, specific microbial signatures linked to post-weaning diarrhea remain incompletely defined. In this case–control study, we performed 16S rRNA gene amplicon sequencing to characterize and compare fecal microbiota of 101 diarrheic and 101 clinically healthy female Holstein calves at 7 days post-weaning. Diarrheic calves exhibited significantly higher species richness (Chao1 index, *p* = 8.7 × 10^−21^) but reduced community evenness (Simpson index, *p* = 2.3 × 10^−10^), a distinct pattern from most neonatal calf diarrhea reports. Microbial community structure differed significantly between groups (PERMANOVA, Bray–Curtis dissimilarity, R^2^ = 0.084, *p* = 0.001). Four genera including Faecousia (fold change = 1.40) were enriched in diarrheic calves, while six genera including Duncaniella (fold change = 0.34) were depleted after FDR correction and validated by complementary statistical methods. PICRUSt2 functional prediction revealed downregulated carbohydrate metabolism and energy biosynthesis pathways in diarrheic calves, alongside upregulated xenobiotic degradation and stress response pathways. This study identifies high-confidence bacterial biomarkers for post-weaning calf diarrhea, providing a solid microbial basis for developing targeted microbiota-based prevention and intervention strategies.

## 1. Introduction

Calf health during the weaning transition is a critical determinant of lifetime growth performance and production efficiency in dairy cattle systems. Post-weaning diarrhea is one of the most common gastrointestinal diseases in intensively raised calves, with reported morbidity ranging from 20 to 50% in commercial herds worldwide [1]. The disease reduces feed intake, impairs growth rate, increases mortality, and drives widespread prophylactic antimicrobial use, which raises concerns about antimicrobial resistance and food safety [2,3]. The pathogenesis of post-weaning diarrhea is multifactorial, involving weaning stress, abrupt dietary shifts, opportunistic pathogen colonization, and immature intestinal immunity [4,5]. Conventional antimicrobial-based treatments have diminishing efficacy and carry public health risks, creating an urgent demand for alternative, microbiota-targeted prevention and control strategies [1,6].

The gastrointestinal tract harbors a complex and dynamic microbial ecosystem that plays indispensable roles in nutrient digestion, intestinal barrier maintenance, immune system maturation, and colonization resistance against pathogens [7,8]. In young calves, the fecal microbiota undergoes rapid ecological succession during the first months of life and is highly vulnerable to dietary and environmental perturbations at weaning [9,10]. Accumulating evidence links gut microbial dysbiosis directly to the onset and progression of diarrhea in young livestock. For example, diarrheic neonatal calves typically show restructured microbial communities, altered commensal populations, and disrupted metabolic function [11,12,13]. These findings support the concept that restoring microbial homeostasis is a promising avenue for managing calf diarrhea.

However, existing research on calf fecal microbiota has focused largely on neonatal diarrhea or dynamic microbial succession during the weaning transition itself [14,15]. Few studies have systematically characterized the fecal microbiota of calves with persistent diarrhea after weaning is complete—a stage when dietary composition has stabilized and acute weaning stress has partially subsided. Furthermore, reported microbial signatures of calf diarrhea vary considerably across herds, breeds, and production systems, and the specific bacterial taxa linked to post-weaning diarrhea remain poorly defined [11,16]. Without a validated profile of diarrhea-associated microbiota, the rational design of precise probiotic or nutritional interventions remains challenging.

In the present study, we performed 16S rRNA gene sequencing to compare fecal microbiota composition and predicted functional capacity of diarrheic versus healthy Holstein calves at 7 d post-weaning. The primary objectives were to quantify differences in fecal microbial diversity and community structure between post-weaning diarrheic and healthy calves, identify key bacterial taxa associated with diarrhea occurrence, and predict functional pathways through which microbiota dysbiosis may contribute to diarrhea pathogenesis. Our findings provide fundamental microbial data and a theoretical framework for developing targeted, microbiota-based intervention strategies against post-weaning diarrhea in dairy calves.

## 2. Materials and Methods

### 2.1. Experimental Design and Fecal Sample Collection

This was an observational case–control study conducted on a commercial dairy farm in Hohhot, China. The experimental unit was the individual calf. A study protocol, including the research question, key design features, and the analysis plan, was prepared before sample collection; the protocol was not registered in a public registry.

Calves were housed individually in hutches within the same barn; calves of both groups were co-housed in this barn and shared the same airspace, with no physical separation imposed between the groups at the barn level. All calves were female Holstein calves born to primiparous dams, weaned at 67 days of age, and fed according to the farm’s standard protocol (milk replacer 8 L/day before weaning; starter feed and chopped hay ad libitum after weaning; fresh water available ad libitum). Bedding consisted of straw, renewed every 7 d. Animals were inspected by farm staff at least 3 times daily; calves with abnormal feces were examined and treated by a veterinarian according to the farm’s protocol. Calves receiving antibiotics within 2 weeks before sampling were excluded. No adverse events were observed during the study, and no humane endpoints were required because no experimental interventions were administered. Calves were housed in individual hutches within the same barn, and diarrheic and healthy calves were co-housed under identical feeding and management conditions; no physical separation was imposed between groups. Healthy Holstein calves born to primiparous dams (all first parity and all female) with comparable birth weights were selected and reared under uniform feeding and management conditions. Birth weight did not differ significantly between groups (healthy: 36.85 ± 0.46 kg; diarrheic: 36.61 ± 0.51 kg; *p* > 0.05). Calves were weaned at 67 days of age, and fecal samples were collected at 7 days post-weaning. Calves were assigned to two groups according to clinical fecal scores assessed by two trained veterinarians using a standardized 4-point scoring system: score 1 = firm, well-formed feces; score 2 = soft, formed feces; score 3 = loose, semi-liquid feces; score 4 = watery, liquid feces with no solid content. The healthy control group included calves with a fecal score of 1–2 (no diarrhea symptoms, normal formed feces), and the post-weaning diarrheic group included calves with a fecal score of 3–4 (watery or loose feces, persistent diarrhea for ≥24 h). Fecal consistency was scored independently by two observers, and discordant scores were resolved by consensus (inter-observer agreement κ = 0.87). No antibiotic administration within 2 weeks prior to sampling was an exclusion criterion for both groups. A total of 202 calves were enrolled in this study. The sample size was determined a priori using G*Power (version 3.1.9.7). Based on previous 16S rRNA studies comparing fecal microbiota between diarrheic and healthy calves, which typically reported effect sizes (Cohen’s d) of 0.6–0.9 for key alpha diversity indices and differentially abundant genera, a two-sided two-group comparison with α = 0.05, power (1 − β) = 0.90, and a target effect size of d = 0.6 required a minimum of 59 animals per group. We enrolled 101 diarrheic and 101 healthy calves to provide robust power with allowance for potential sample loss during DNA extraction and sequencing. Post-hoc power analysis using the observed effect sizes confirmed that the achieved power exceeded 0.95 for all significantly different alpha diversity indices and differential genera. Fresh fecal samples were collected directly from the rectum using sterile disposable gloves, immediately snap-frozen in liquid nitrogen, and transferred to a −80 °C ultra-low freezer (Thermo Fisher Scientific, Waltham, MA, USA) for long-term storage until microbial DNA extraction. All animal procedures in this study were approved by the Institutional Animal Care and Use Committee of Hetao College (Approval No. STX0314).

### 2.2. Microbial DNA Extraction and 16S rRNA Gene Amplicon Sequencing

Total genomic DNA was extracted from approximately 200 mg of each fecal sample using the QIAamp Fast DNA Stool Mini Kit (Qiagen, Hilden, Germany) strictly following the manufacturer’s protocol, with the addition of a bead-beating step (0.5 g of 0.1 mm zirconia beads, 30 Hz for 2 × 3 min) to enhance lysis of Gram-positive bacteria. One extraction blank control (no fecal material) was included for every batch of 24 extractions to monitor reagent contamination. The concentration and purity of extracted DNA were determined with a NanoDrop 2000 spectrophotometer (Thermo Fisher Scientific, Wilmington, DE, USA), and DNA concentration was further verified fluorometrically using the Qubit dsDNA HS Assay Kit (Thermo Fisher Scientific, Waltham, MA, USA). DNA integrity was verified by 1% agarose gel electrophoresis. The V3–V4 hypervariable region of the bacterial 16S rRNA gene was amplified via PCR using the universal primer pair 338F (5′-ACTCCTACGGGAGGCAGCAG-3′) and 806R (5′-GGACTACHVGGGTWTCTAAT-3′), with sample-specific 8 bp barcodes attached to the 5′ end of the forward primer for multiplexing. PCR reactions were performed in a 20 μL reaction system containing 4 μL of 5× FastPfu Buffer, 2 μL of 2.5 mM dNTPs, 0.8 μL of each primer (5 μM), 0.4 μL of FastPfu Polymerase, and 10 ng of template DNA. Three technical PCR replicates were performed per sample and pooled to reduce PCR stochasticity. A PCR negative control (no template DNA) was included in each amplification batch. The thermal cycling program was set as follows: initial denaturation at 95 °C for 3 min; 27 cycles of denaturation at 95 °C for 30 s, annealing at 55 °C for 30 s, and extension at 72 °C for 45 s; and a final extension at 72 °C for 10 min. PCR amplicons were purified using the AxyPrep DNA Gel Extraction Kit (Axygen Biosciences, Union City, CA, USA) and quantified with a Quantus™ Fluorometer (Promega, Madison, WI, USA). Purified amplicons were pooled in equimolar amounts and paired-end high-throughput sequencing (2 × 250 bp) was carried out on the Illumina NovaSeq 6000 platform (Illumina, San Diego, CA, USA), with a 10% PhiX control library spike-in to improve base-calling accuracy.

The average number of raw paired-end reads per sample was 83,452 ± 12,307 (mean ± SD), and no contamination was detected in extraction or PCR negative controls.

### 2.3. Bioinformatic Analysis

Bioinformatic analyses of microbiome data were conducted using QIIME 2 (version 2019.4). Raw sequencing reads were demultiplexed with the demux plugin. Primer sequences were trimmed with the cutadapt plugin (qiime cutadapt trim-paired) using the forward primer 338F (5′-ACTCCTACGGGAGGCAGCAG-3′) and the reverse primer 806R (5′-GGACTACHVGGGTWTCTAAT-3′), with a maximum error rate of 0.1. Quality filtering, sequence denoising, paired-end read merging, and chimera removal were performed with the DADA2 plugin (qiime dada2 denoise-paired). After quality filtering and denoising, the average number of high-quality reads per sample was 68,234 ± 9876 (mean ± SD; range: 42,105–91,342). For alpha and beta diversity calculations, the ASV table was rarefied to a depth of 25,000 reads per sample. The rarefaction curves (Supplementary Figure S1) for both the healthy and diarrhea groups approached saturation at this sequencing depth, demonstrating that the current sequencing depth adequately recovered most microbial ASVs within the samples. Multiple sequence alignment of ASV representative sequences was conducted using MAFFT, and a phylogenetic tree was constructed with FastTree 2. Alpha diversity indices (Chao1, observed species, Shannon, Gini–Simpson, Faith’s phylogenetic diversity, Pielou’s evenness, and Good’s coverage) and beta diversity metrics (weighted UniFrac, unweighted UniFrac, Jaccard distance, and Bray–Curtis dissimilarity) were calculated with the diversity plugin. Taxonomic classification of ASVs was performed with the feature-classifier plugin using the classify-sklearn naïve Bayes classifier (confidence threshold [0.7]), trained on the V3–V4 region (338F/806R) of the SILVA 138.1 (99% OTU) reference; the taxon names displayed follow the GTDB release 202 nomenclature, applied as described below. The functional potential of the fecal microbiota was predicted using PICRUSt2 (version 2.5.0). Predicted EC gene families were mapped to MetaCyc pathway abundances (pathway_pipeline.py, default parameters), and the resulting pathway identifiers (PWY-style) are those displayed in Results 3.6; predicted KO gene families were mapped to KEGG orthologs via the EC-to-KO mapping. Predicted metagenome abundances were normalized by predicted 16S rRNA gene copy number. The nearest sequenced taxon index (NSTI) values were calculated to assess prediction reliability (mean NSTI = 0.12 ± 0.04, range; default cutoff of 2). It should be noted that PICRUSt2 predicts functional potential based on 16S rRNA gene phylogeny and does not directly measure gene expression or metabolic activity.

### 2.4. Statistical Analysis

All statistical analyses were performed using R software (version 4.2.0) and GraphPad Prism (version 10.0; GraphPad Software, La Jolla, CA, USA). The normality of data distribution was tested via the Shapiro–Wilk test. For alpha diversity indices, intergroup differences between healthy and diarrheic calves were evaluated using the Mann–Whitney U test (nonparametric test). Beta diversity patterns were visualized by principal coordinate analysis (PCoA) based on Bray–Curtis and UniFrac distances, and the statistical significance of community structure differences between groups was assessed by permutational multivariate analysis of variance (PERMANOVA; vegan::adonis2 in R 4.2.0, model formula “distance ~ group”, 999 permutations, set.seed(42)). Differences in multivariate dispersion were tested with PERMDISP (vegan::betadisper followed by permutest, 999 permutations). Non-metric multidimensional scaling (NMDS) was performed with vegan::metaMDS using Bray–Curtis dissimilarity, k = 2 dimensions, trymax = 20, and set.seed(42). Differential abundance at the genus level was assessed using the Mann–Whitney U test on genus-level relative abundances derived from the rarefied ASV table (30,000 reads per sample), with zero values retained as zeros. Genera with a mean relative abundance > 0.1% in at least one group and present in ≥20% of samples in at least one group were tested. *p*-values were adjusted for multiple comparisons using the Benjamini–Hochberg false discovery rate (FDR) method, and the resulting q-values are reported in Table 1. Fold change (FC) was calculated as the ratio of the mean relative abundance in the diarrheic group to that in the healthy group, and its 95% confidence interval was estimated by bootstrap resampling (10,000 resamples). The detailed statistical results of differential genera are summarized in Table 1. For alpha and beta diversity calculations, the ASV table was rarefied to a depth of 30,000 reads per sample (the minimum post-filtering read count among all samples was 42,105 reads; 30,000 reads was selected as the rarefaction depth because it is below the minimum observed depth and thus retained all 202 samples). Differentially abundant taxa between groups were identified using two complementary methods: (1) the linear discriminant analysis effect size (LEfSe) method, with an LDA score threshold of 2.0 and a significance level of *p* < 0.05; and (2) ANCOM-BC (Analysis of Composition of Microbiomes with Bias Correction), a compositionality-aware method that accounts for the compositional nature of microbiome data, with an FDR-adjusted significance threshold of *p* < 0.05. Taxa identified as significantly differential by both methods were reported as high-confidence differential taxa. Differential abundance at the genus level was assessed using the Mann–Whitney U test, with *p*-values adjusted for multiple comparisons using the Benjamini–Hochberg false discovery rate (FDR) method. Fold change (FC) was calculated as the ratio of the mean relative abundance in the diarrheic group to that in the healthy group. Differentially abundant taxa between groups were identified using the linear discriminant analysis effect size (LEfSe) method, with a linear discriminant analysis (LDA) score threshold of 2.0 and a significance level of *p* < 0.05. LEfSe internally employs a Kruskal–Wallis rank-sum test (nominal *p* < 0.05) followed by LDA for effect size estimation. To account for multiple comparisons and the large sample size (*n* = 202), we additionally applied Benjamini–Hochberg FDR correction across all tested features; only taxa with FDR-adjusted *p* < 0.05 and LDA score > 2.0 were reported. Differences in predicted KEGG functional pathways were compared using the Mann–Whitney U test, and *p*-values were adjusted via the Benjamini–Hochberg false discovery rate (FDR) method. A *p*-value < 0.05 was considered statistically significant for all analyses.

## 3. Results

### 3.1. Alterations in Fecal Microbiota Alpha Diversity

To evaluate the effect of diarrhea on fecal microbial richness and diversity in post-weaning calves, alpha diversity indices were calculated on a rarefied ASV table (30,000 reads per sample), and four classic alpha diversity metrics were quantified: Chao1, observed species, Shannon index, and Simpson index. As shown in Figure 1a, the diarrheic group exhibited significantly higher microbial richness than the healthy group, supported by both the Chao1 index (1066.49 vs. 809.96, *p* = 8.7 × 10^−21^) and the number of observed species (1215.77 vs. 790.58, *p* = 1.6 × 10^−20^). For community diversity, the Shannon index was markedly elevated in diarrheic calves compared with healthy controls (8.50 vs. 7.69, *p* = 1.2 × 10^−15^). In contrast, the Simpson index was significantly lower in the diarrheic group (0.987 vs. 0.980, *p* = 2.3 × 10^−10^), indicating reduced community evenness despite increased species richness. All four indices reached extremely significant differences between groups (all *p* < 0.001), demonstrating that diarrhea profoundly reshaped the richness, diversity, and evenness patterns of the fecal microbial community in post-weaning calves.

### 3.2. Shifts in Fecal Microbial Community Structure

Beta diversity analyses were performed to characterize the overall structural differentiation of fecal microbiota between healthy and diarrheic calves. PERMANOVA with 999 permutations revealed significant differences in community structure between groups for all four distance metrics: Bray–Curtis (F = 18.34, R^2^ = 0.084, *p* = 0.001), weighted UniFrac (F = 15.67, R^2^ = 0.072, *p* = 0.001), unweighted UniFrac (F = 22.15, R^2^ = 0.099, *p* = 0.001), and Jaccard (F = 16.89, R^2^ = 0.078, *p* = 0.001). PERMDISP analysis confirmed no significant difference in multivariate dispersion between groups for any metric (all *p* > 0.05, Supplementary Table S1), indicating that the observed separation reflects true differences in community composition rather than differences in within-group variability. Principal Coordinates Analysis (PCoA) based on Bray–Curtis dissimilarity revealed a clear separation of microbial communities along the first principal coordinate (Figure 1b). PCo1 explained 11.2% of the total variance and served as the main axis driving group differentiation, while PCo2 accounted for 4.3% of the variance.

Non-metric Multidimensional Scaling (NMDS) was further applied to verify the structural difference. Consistent with PCoA results, NMDS ordination showed that samples from the healthy and diarrheic groups formed distinct clusters with negligible overlap (Figure 1c). The stress value of NMDS was 0.128, which is below the 0.2 threshold and confirms the reliability of the two-dimensional ordination. Collectively, both beta diversity approaches consistently demonstrated that diarrhea induced a significant structural shift in the gut microbial community of post-weaning calves.

### 3.3. Taxonomic Composition of Fecal Microbiota

To systematically dissect the fecal microbial community structure of post-weaning calves at different taxonomic levels, we first profiled the relative abundance of microbiota at the phylum level. As shown in Figure 2a, Firmicutes_A and Bacteroidota were the two absolutely dominant phyla in both healthy and diarrheic calves, with a combined mean relative abundance of 86.22% in the healthy group (Firmicutes_A: 45.82%, Bacteroidota: 40.40%) and 90.52% in the diarrheic group (Firmicutes_A: 55.05%, Bacteroidota: 35.47%). Mann–Whitney U tests with FDR correction revealed that the diarrheic group exhibited a significant increase in the relative abundance of Firmicutes_A (55.05% vs. 45.82%, FDR-adjusted *p* = 2.3 × 10^−6^), while the proportion of Bacteroidota was significantly decreased (35.47% vs. 40.40%, FDR-adjusted *p* = 1.1 × 10^−4^). For subdominant phyla, Firmicutes_D showed an elevated abundance in the diarrheic group (4.09%) relative to the healthy group (2.75%, FDR-adjusted *p* = 0.012), whereas all other phyla, including Proteobacteria, Spirochaetota, Verrucomicrobiota, Actinobacteriota, Desulfobacterota_I, and Firmicutes_B, exhibited varying degrees of reduction in relative abundance in the diarrheic group (all FDR-adjusted *p* < 0.05). Compared with the healthy group, the diarrheic group exhibited a significant increase in the relative abundance of Firmicutes_A (55.05% vs. 45.82%), while the proportion of Bacteroidota was markedly decreased (35.47% vs. 40.40%). For subdominant phyla, Firmicutes_D showed an elevated abundance in the diarrheic group (4.09%) relative to the healthy group (2.75%), whereas all other phyla, including Proteobacteria, Spirochaetota, Verrucomicrobiota, Actinobacteriota, Desulfobacterota_I, and Firmicutes_B, exhibited varying degrees of reduction in relative abundance in the diarrheic group. These results demonstrate that diarrhea induced a profound restructuring of the fecal microbial community at the phylum level, with a clear shift in the dominant phylum ratio and a widespread decline in the abundance of subdominant phyla.

At the genus level (Figure 2b), the overall composition profile of dominant genera showed distinct differences between the two groups. *Faecousia* was the most dominant genus in both groups, followed by *Cryptobacteroides*. Genera such as *Faecousia* and *Phocaeicola_A* accounted for a higher proportion in the diarrheic group, while *Cryptobacteroides*, *Alloprevotella* and *Paraprevotella* were more abundant in the healthy group, indicating that diarrhea additionally reshaped the fine structure of the fecal microbial community at the genus level.

### 3.4. Differentially Abundant Genera Associated with Post-Weaning Calf Diarrhea

To further identify key microbial taxa linked to post-weaning calf diarrhea, differential abundance analysis was performed at the genus level. As shown in the sample-level heatmap (Figure 2c), in which each column represents an individual sample, samples clustered predominantly by group, consistent with the beta diversity results. Diarrhea-enriched genera and health-enriched genera were clearly separated, reflecting distinct microbial signatures between the two groups. The detailed statistical results of differential genera are summarized in Table 1. A total of 10 genera showed significant differences between the healthy and diarrheic groups (all *p* < 0.01). Among them, 4 genera were significantly enriched in the diarrheic group, including *Faecousia* (FC = 1.40, *p* < 0.001), *Phocaeicola_A* (FC = 1.84, *p* < 0.001), *Faecalimonas* (FC = 1.88, *p* < 0.001) and *UBA737* (FC = 1.44, *p* < 0.01). Conversely, 6 genera were significantly depleted in the diarrheic group. *Duncaniella* showed the largest reduction (FC = 0.34, *p* < 0.001), followed by *Paraprevotella* (FC = 0.45, *p* < 0.001) and *Alloprevotella* (FC = 0.46, *p* < 0.001). These differential taxa can serve as potential microbial biomarkers for post-weaning calf diarrhea and provide candidate targets for the development of subsequent intervention strategies.

### 3.5. Identification of Diarrhea-Associated Microbial Biomarkers by LEfSe

To further identify key microbial taxa with high discriminative power for post-weaning calf diarrhea, we performed LEfSe analysis with an LDA score threshold of 2.0, complemented by ANCOM-BC analysis to validate differential abundance while accounting for data compositionality. As shown in Figure 3a, a total of 14 taxa with LDA scores > 2 were identified as differential biomarkers between the two groups by LEfSe. Among these, 10 genera were concordantly identified as significantly differentially abundant by ANCOM-BC (FDR-adjusted *p* < 0.05), representing high-confidence differential taxa (summarized in Table 1). The remaining 4 LEfSe-identified taxa (at higher taxonomic ranks) were not independently significant at the genus level by ANCOM-BC and are reported as exploratory findings. Taxa enriched in the healthy group were mainly affiliated with the phylum Bacteroidota, including the class Bacteroidia, order Bacteroidales, family Bacteroidaceae, and genera *Paraprevotella* and *Duncaniella*. In contrast, taxa enriched in the diarrheic group were predominantly derived from the phylum Firmicutes_A, covering the class Clostridia, orders Oscillospirales and Lachnospirales, family Lachnospiraceae, and genera *Faecousia* and *Phocaeicola_A*. Among them, *Faecousia* exhibited the highest LDA score in the diarrheic group, indicating its strong discriminatory ability for diarrheal status.

The cladogram (Figure 3b) intuitively visualized the hierarchical distribution of these differential taxa from phylum to genus level. Consistent with the LDA histogram, differential taxa in the healthy group were concentrated in the Bacteroidota branch, while differential taxa in the diarrheic group were mainly clustered in the Firmicutes_A branch, presenting a clear lineage-specific enrichment pattern. This result further confirms that diarrhea induced a lineage-level structural shift in the fecal microbiota of post-weaning calves. The microbial biomarkers identified by LEfSe can serve as reliable indicators for evaluating the intestinal health status of post-weaning calves and provide precise targets for the development of targeted probiotic or dietary intervention strategies against calf diarrhea.

### 3.6. Functional Alterations of Fecal Microbiota in Diarrheic Calves Predicted by PICRUSt2

To elucidate the functional implications of fecal microbiota dysbiosis in post-weaning calves with diarrhea, we predicted the functional potentials of microbial communities using PICRUSt2 and compared the overall functional profiles between healthy and diarrheic groups. Principal Coordinates Analysis of the functional gene profiles demonstrated a separation of the microbial functional architecture between the two groups (Figure 4a). PERMANOVA on the predicted functional gene family profiles (Bray–Curtis distance, 999 permutations) confirmed a significant difference between the groups and PERMDISP showed no significant difference in dispersion, indicating that the separation reflects a true difference in predicted functional composition rather than within-group variability (Supplementary Table S2). Thus, the predicted functional profiles of the fecal microbiota differed significantly between the two groups, and this difference was associated with diarrhea status. We further performed differential abundance analysis to pinpoint specific functional pathways associated with post-weaning diarrhea. As illustrated in Figure 4b, a total of 28 predicted metabolic pathways (MetaCyc) exhibited significant differences between the two groups (Mann–Whitney U test, FDR-adjusted *p* < 0.05; see Supplementary Table S3 for the complete list with full pathway names, functional categories, and statistical values). Pathways overrepresented in the healthy group were mainly implicated in carbohydrate metabolism, energy generation, and amino acid biosynthesis, whereas pathways overrepresented in the diarrheic group were largely related to xenobiotic biodegradation and stress response. These differences in predicted functional potential were consistent with the taxonomic differences observed at the phylum and genus levels, and provided functional evidence for the involvement of fecal microbiota dysbiosis in the pathogenesis of post-weaning calf diarrhea, offering potential functional targets for subsequent intervention strategies.

## 4. Discussion

In this study, we comprehensively profiled the fecal microbiota of post-weaning diarrheic and healthy dairy calves. Our core finding is that post-weaning diarrhea in calves is associated with profound fecal microbiota dysbiosis, manifesting as altered diversity patterns, restructured community composition, lineage-specific taxonomic shifts, and disrupted metabolic functions. These results reinforce the consensus that microbial imbalance is a hallmark of post-weaning gastrointestinal disorders in young ruminants [5,17].

### 4.1. Diversity and Community Structure Alterations

A striking observation in our study is that diarrheic calves showed increased species richness (Chao1 and observed species) but reduced community evenness (Simpson index) relative to healthy controls. This pattern differs from some neonatal diarrhea studies that report uniformly reduced alpha diversity, and may reflect the specific pathophysiological stage of post-weaning diarrhea [12,18]. In the early to mid-stage of diarrhea, inflammatory intestinal conditions can create new ecological niches that allow proliferation of multiple opportunistic and conditionally tolerant taxa, thereby increasing observed species richness [19,20]. At the same time, a small number of dominant genera expand disproportionately, reducing overall community evenness and destabilizing the microbial ecosystem. Reduced evenness is widely recognized as a marker of ecosystem instability and reduced resistance to further perturbation [9].

Beta diversity analysis further confirmed a clear structural separation of microbial communities between diarrheic and healthy calves. This indicates that post-weaning diarrhea is associated with a fundamental restructuring of the gut microbiome rather than minor taxonomic shifts, consistent with previous observations in pre-weaning calf diarrhea and weaned piglet diarrhea models [9,11].

### 4.2. Taxonomic Shifts Associated with Diarrhea

At the phylum level, we observed an increased ratio of Firmicutes_A to Bacteroidota in diarrheic calves. An elevated Firmicutes-to-Bacteroidetes ratio has been linked to intestinal inflammation and altered energy harvest in multiple gastrointestinal disorders [21,22]. The expansion of Firmicutes_A, particularly families within Clostridia, may reflect adaptive proliferation of bacteria tolerant to inflammatory gut environments.

At the genus level, *Faecousia*, *Phocaeicola_A*, and *Faecalimonas* were significantly enriched in diarrheic calves. *Faecousia*, a member of the Lachnospiraceae family, has been reported to increase in abundance under intestinal inflammatory conditions and may participate in mucus degradation [23]. Enrichment of *Phocaeicola_A*, a genus within Bacteroidaceae, has also been associated with disrupted gut barrier function and inflammatory responses in ruminants [12]. It should be noted that *Faecousia* and *Phocaeicola_A* are relatively recently delineated genera in the GTDB classification system, and the existing literature on their specific functional roles remains limited, with most evidence coming from single recent studies. *Faecousia*, a member of the Lachnospiraceae family, has been reported in one study to increase in abundance under intestinal inflammatory conditions and may be associated with mucus degradation, though this requires further validation. Enrichment of *Phocaeicola_A*, a genus within Bacteroidaceae, has also been associated with disrupted gut barrier function and inflammatory responses in ruminants in a single report. These associations suggest that the overgrowth of these taxa may reflect their competitive advantage in the inflamed post-weaning gut environment, although further studies are needed to determine whether these taxa contribute to diarrhea pathogenesis or merely proliferate as a consequence of the altered intestinal environment. First, this was an observational cross-sectional study in which microbiota and diarrhea status were assessed simultaneously; therefore, the directionality of the observed associations cannot be established—microbiota dysbiosis may be a cause, a consequence, or both in relation to diarrhea. Longitudinal studies with pre-diarrhea sampling are needed to establish temporal relationships. Conversely, multiple genera with known beneficial functions were significantly depleted in diarrheic calves, including *Alloprevotella*, *Paraprevotella*, *Prevotella*, and *Duncaniella*. Members of *Prevotella* and *Alloprevotella* are major degraders of complex dietary carbohydrates and important producers of short-chain fatty acids (SCFAs) in the calf gut [24,25]. SCFAs serve as the primary energy source for colonocytes, support tight junction integrity, and exert anti-inflammatory effects [26,27]. Depletion of these SCFA-producing commensals in diarrheic calves implies impaired fermentative capacity and reduced intestinal barrier support, which may exacerbate diarrhea severity. The reduction in *Duncaniella*, a genus associated with stable gut homeostasis, further indicates loss of key homeostatic taxa in the diarrheic state [16]. The microbial signatures identified here provide a rational basis for selecting candidate probiotics, but extensive validation is required before clinical or agricultural application.

### 4.3. Functional Implications of Microbiota Dysbiosis

In line with taxonomic shifts, functional prediction analysis revealed markedly different metabolic profiles between groups. Pathways involved in carbohydrate metabolism and energy biosynthesis were downregulated in diarrheic calves, consistent with depletion of carbohydrate-degrading and SCFA-producing commensals [15,21]. Diminished capacity for carbohydrate fermentation reduces energy supply to intestinal epithelial cells, impairs barrier integrity, and diminishes anti-inflammatory signaling in the gut.

Meanwhile, pathways related to xenobiotic degradation and stress response were upregulated in the diarrheic group. This functional reprogramming reflects adaptive responses of the gut microbial community to the altered intestinal environment during diarrhea, including increased exposure to inflammatory mediators and metabolic stress [19,20]. These functional alterations provide mechanistic insight into how microbiota dysbiosis contributes to diarrhea pathogenesis in post-weaning calves.

### 4.4. Limitations

Several limitations of this study should be acknowledged. First, we did not perform etiological testing for common diarrheal pathogens (e.g., rotavirus, coronavirus, Cryptosporidium, Escherichia coli, Salmonella). Therefore, we cannot determine whether the observed microbial shifts are a direct consequence of specific pathogen infection or a general feature of post-weaning diarrhea irrespective of etiology. Future studies should integrate multiplex pathogen detection with 16S rRNA sequencing to disentangle pathogen-specific and nonspecific microbial signatures. Second, this is an observational cross-sectional study; causality between microbiota dysbiosis and diarrhea cannot be established. All calves were sourced from a single farm and a single birth cohort, which may limit the generalizability of the findings to other farms, breeds, geographic regions, or seasonal conditions. Multi-farm, multi-cohort studies are needed to validate the microbial signatures identified here. Third, PICRUSt2 provides predicted functional potentials rather than measured gene expression, and metabolomic validation is warranted in future work.

## 5. Conclusions

This study demonstrates that post-weaning diarrheic dairy calves exhibit significant fecal microbiota dysbiosis, characterized by increased species richness, reduced community evenness, lineage-specific taxonomic shifts, and disrupted metabolic functions. We identified key bacterial taxa strongly associated with post-weaning diarrhea status, including enriched genera such as *Faecousia* and depleted SCFA-producing commensals such as *Alloprevotella* and *Paraprevotella*. These findings advance understanding of the microbial basis of post-weaning diarrhea in calves and provide a potential microbial reference for the rational development of effective, microbiota-based prevention and treatment strategies.

## Figures and Tables

**Figure 1:**
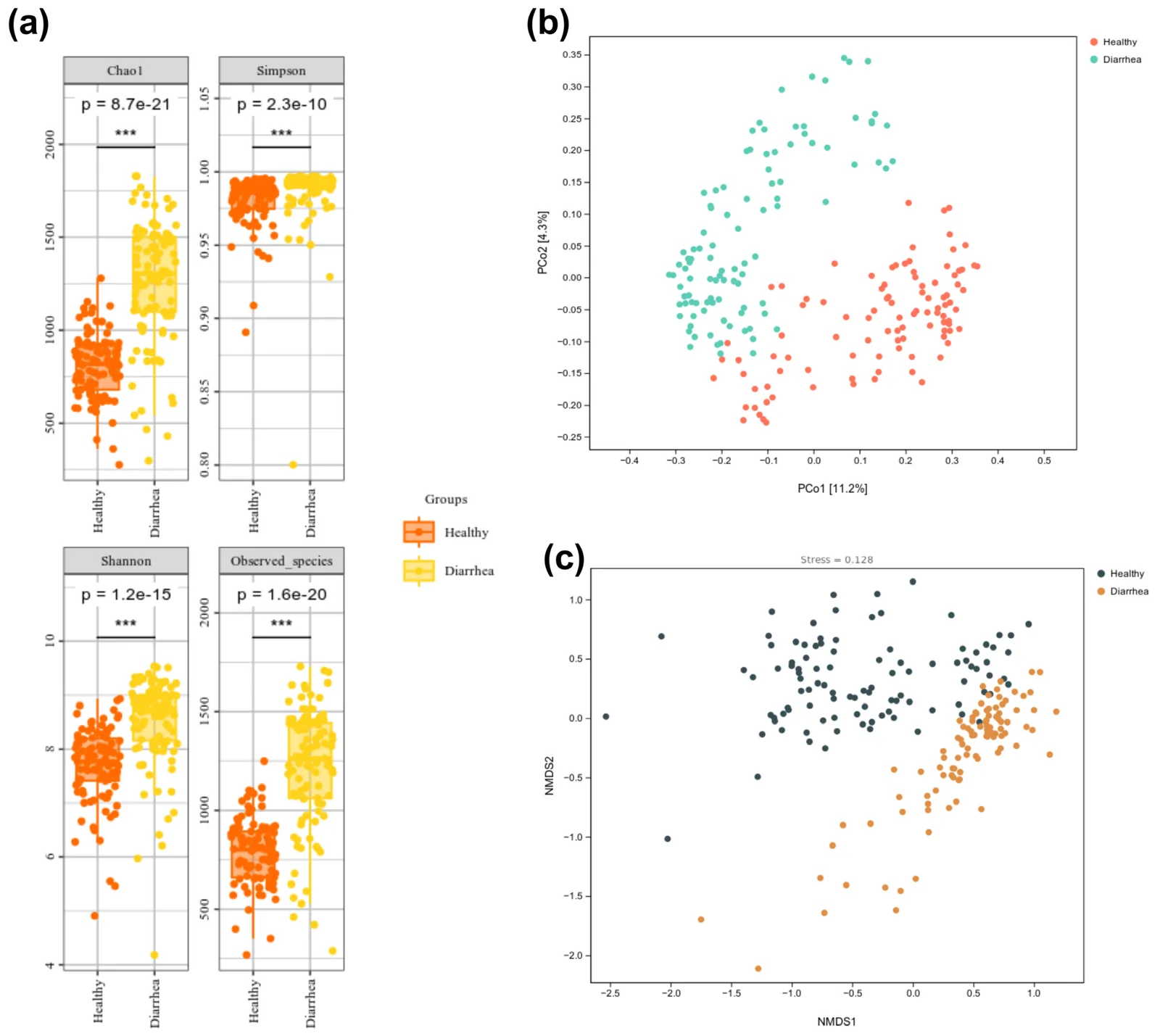
Comparison of gut microbial diversity between healthy and diarrheic post-weaning calves: (**a**) Alpha diversity profiles of fecal microbiota, including Chao1 index, Simpson index, Shannon index, and observed species number. Orange boxes/dots represent the healthy group, and yellow boxes/dots represent the diarrheic group. Statistical significance was assessed by Wilcoxon rank-sum test (*** *p* < 0.001). (**b**) Principal Coordinates Analysis (PCoA) based on Bray–Curtis dissimilarity illustrating the structural separation of gut microbial communities between the healthy (red dots) and diarrheic (teal dots) groups. The first and second axes explain 11.2% and 4.3% of the total variance, respectively. (**c**) Non-metric multidimensional scaling (NMDS) ordination based on Bray–Curtis dissimilarity showing the clustering patterns of the healthy (dark green dots) and diarrheic (orange dots) groups. The stress value of NMDS is 0.128, indicating a good fit of the ordination.

**Figure 2:**
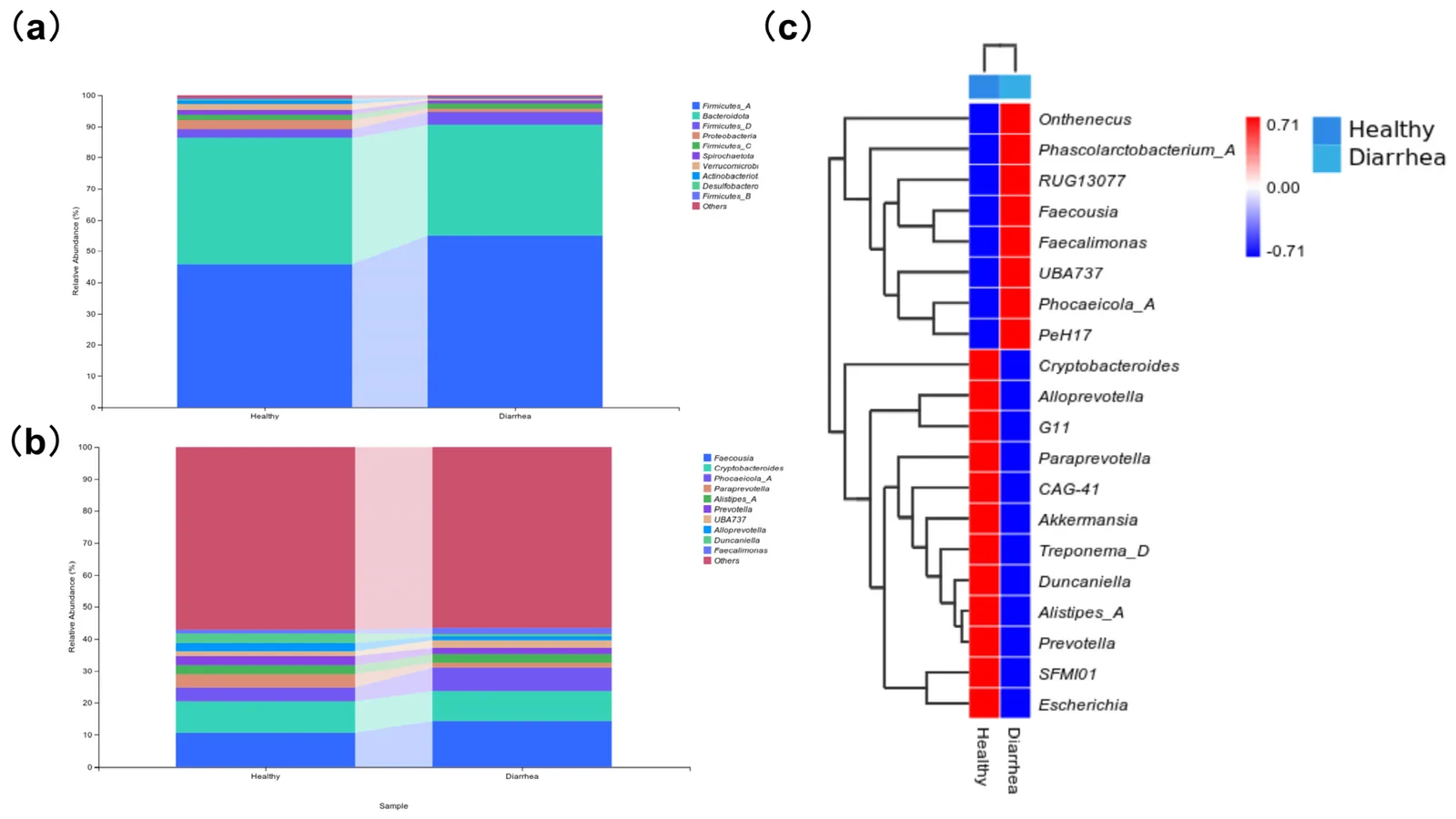
Taxonomic composition and differential taxa analysis of fecal microbiota between healthy and diarrheic post-weaning calves: (**a**) Stacked bar chart illustrating the relative abundance of fecal microbiota at the phylum level in the healthy and diarrheic groups. The top 10 phyla are displayed, with the remaining taxa merged into the “Others” category. (**b**) Stacked bar chart showing the relative abundance of fecal microbiota at the genus level in the healthy and diarrheic groups. The dominant genera with relative abundance > 1% are presented, and low-abundance genera are combined into “Others”. (**c**) Heatmap of differentially abundant genera between the two groups. Columns represent the mean relative abundance of the healthy group and diarrheic group, respectively; rows represent differentially enriched genera, with hierarchical clustering performed based on abundance profiles.

**Figure 3:**
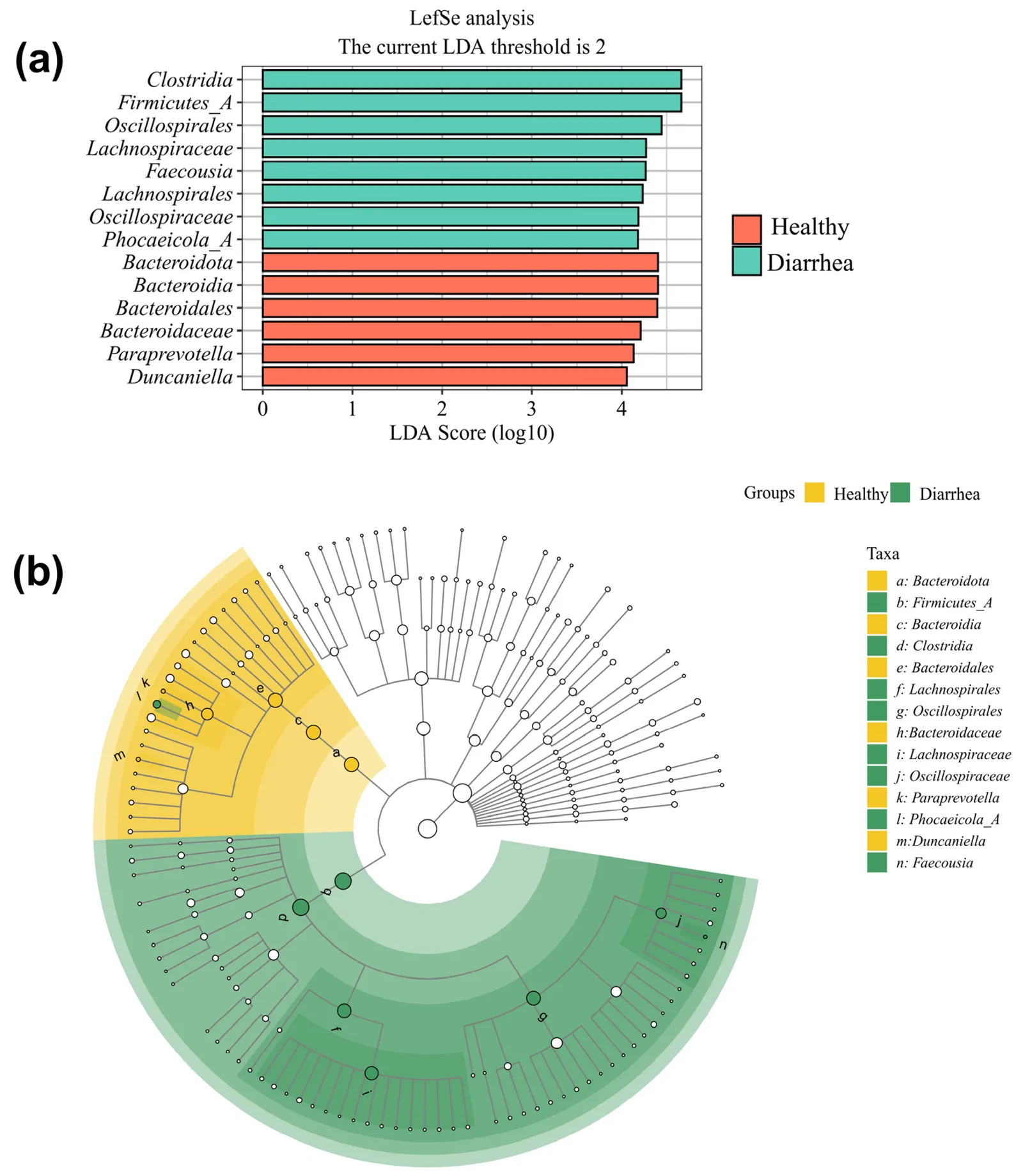
LEfSe analysis of differential microbial biomarkers between healthy and diarrheic post-weaning calves: (**a**) Histogram of linear discriminant analysis (LDA) scores for differentially enriched taxa. The LDA threshold was set at 2.0. Taxa enriched in the healthy group are shown in red, and taxa enriched in the diarrheic group are shown in teal. (**b**) Cladogram showing the hierarchical distribution of differential taxa from phylum to genus level. Yellow nodes represent taxa significantly enriched in the healthy group, and green nodes represent taxa significantly enriched in the diarrheic group. Each circular node corresponds to a taxon at a specific taxonomic level, with the diameter proportional to the relative abundance of the taxon.

**Figure 4:**
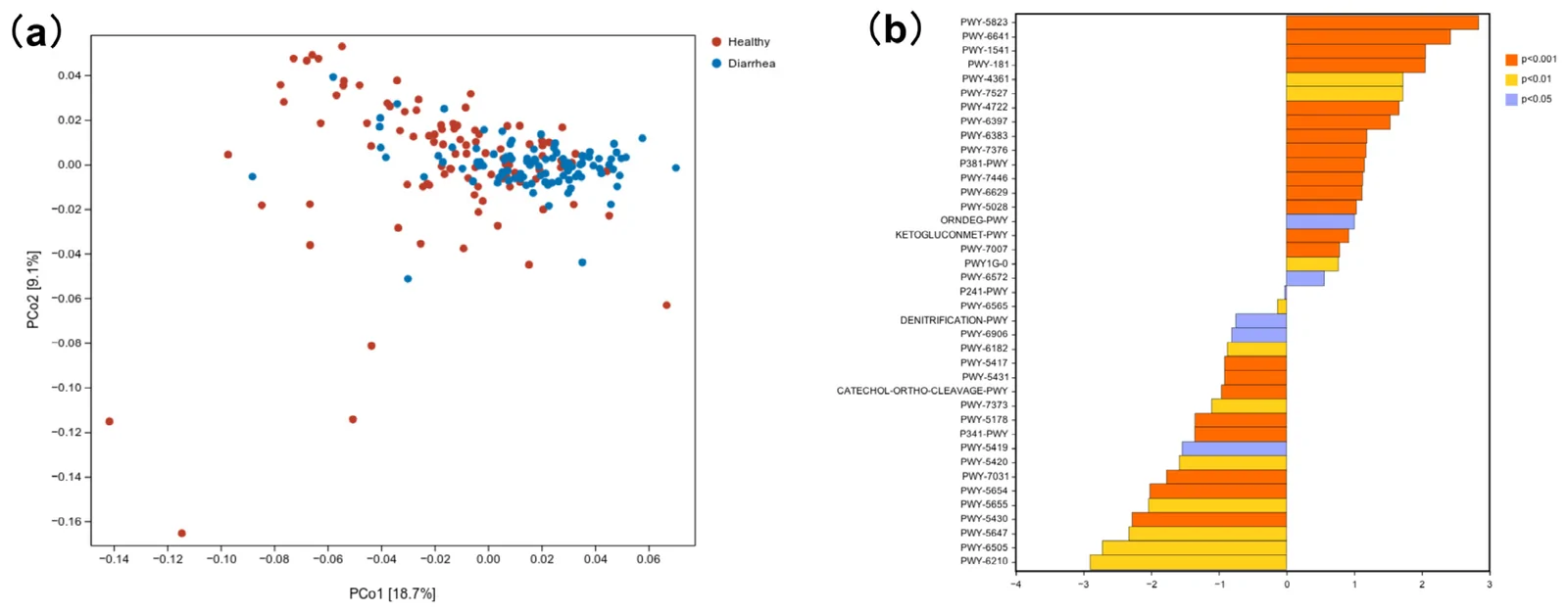
Predicted functional profiles of fecal microbiota between healthy and diarrheic post-weaning calves based on PICRUSt2 analysis: (**a**) Principal Coordinates Analysis (PCoA) of microbial functional gene profiles. Each dot represents an individual sample; red dots correspond to the healthy group and blue dots correspond to the diarrheic group. The percentages in parentheses on the axes indicate the proportion of total variance explained by each principal coordinate. (**b**) Bar chart showing differentially abundant functional pathways between the healthy and diarrheic groups. The *x*-axis represents log_2_(fold change) (Healthy relative to Diarrhea). Positive values indicate pathways enriched in the healthy group, whereas negative values indicate pathways enriched in the diarrheic group. Color coding denotes significance levels based on FDR-adjusted *p*-values: orange for q < 0.001, yellow for q < 0.01, and blue for q < 0.05.

**Table 1:** Fecal microbiota with significant differences between diarrheic and healthy post-weaning calves at the genus level.

Genus	Mean (%)		FC (Diarrhea/Healthy)	*p*-Value
	Diarrhea	Healthy		
*Faecousia*	14.23	10.15	1.40 *** ↑	0.00021
*Phocaeicola_A*	7.85	4.26	1.84 *** ↑	0.00035
*Faecalimonas*	2.87	1.53	1.88 *** ↑	0.00012
*UBA737*	2.54	1.76	1.44 ** ↑	0.0042
*Cryptobacteroides*	7.62	10.89	0.70 *** ↓	0.00058
*Alloprevotella*	1.89	4.12	0.46 *** ↓	0.00017
*Paraprevotella*	1.56	3.47	0.45 *** ↓	0.00032
*Prevotella*	2.04	3.75	0.54 *** ↓	0.0021
*Alistipes_A*	2.11	3.08	0.68 ** ↓	0.0067
*Duncaniella*	1.12	3.29	0.34 *** ↓	0.00089

Notes: *** *p* < 0.001; ** *p* < 0.01. FC, fold change (diarrhea/healthy). Values are Benjamini–Hochberg FDR-adjusted *p*-values (q-values). FC, fold change (diarrhea/healthy); 95% CI, bootstrap confidence interval of FC (10,000 resamples). ↑ indicates enrichment in the diarrheic group; ↓ indicates depletion in the diarrheic group. Taxonomic names follow the GTDB r202 classification system; “_A” suffixes denote distinct GTDB-delineated clades.

## Data Availability

The 16S rRNA gene sequencing datasets generated and analyzed during the current study are available in the NCBI Sequence Read Archive (SRA) under the BioProject accession number PRJNA1522588 (https://www.ncbi.nlm.nih.gov/sra/PRJNA1522588, accessed on 3 September 2026). The processed ASV feature table, taxonomic classification results, sample-to-SRA mapping, complete metadata, rarefaction curves, and all bioinformatic analysis scripts have been deposited in a general-purpose repository (Zenodo, DOI: 10.5281/zenodo.22711064).

## Supplementary Materials

The following supporting information can be downloaded at: https://www.mdpi.com/article/10.3390/microorganisms14102171/s1, Figure S1: Rarefaction curves of amplicon sequence variants (ASVs) for healthy and diarrhea groups. The x-axis represents the sequencing depth (number of reads per sample), and the y-axis shows the observed number of ASVs. The yellow line indicates healthy samples, while the green line represents diarrhea samples. The curves of both groups gradually plateaued at higher sequencing depths, indicating that the sequencing depth was sufficient to recover most microbial taxa in the samples; Table S1: PERMANOVA and PERMDISP results for fecal bacterial community beta-diversity between healthy and diarrheic post-weaning calves; Table S2: PERMANOVA and PERMDISP results for predicted MetaCyc functional profiles of fecal microbiota (PICRUSt2); Table S3: Significantly different MetaCyc pathways between healthy and diarrheic post-weaning calves predicted by PICRUSt2 (corresponding to Figure 4b).

## Abbreviations

ASV	amplicon sequence variant
DADA2	divisive amplicon denoising algorithm 2
FDR	false discovery rate
KEGG	Kyoto Encyclopedia of Genes and Genomes
LEfSe	linear discriminant analysis effect size
NMDS	non-metric multidimensional scaling
PCoA	principal coordinate analysis
PERMANOVA	permutational multivariate analysis of variance
PICRUSt2	phylogenetic investigation of communities by reconstruction of unobserved states 2
SCFA	short-chain fatty acid
