# Supplementary material for: Fecal Microbiota Dysbiosis and Diarrhea-Associated Bacterial Taxa in Post-Weaning Dairy Calves
Source: Microorganisms. 2026 Sep 25;14(10):2171. doi: 10.3390/microorganisms14102171 (PMC13707477; doi:10.3390/microorganisms14102171)
Supplement: Supplementary file 1 [file microorganisms-14-02171-s001.zip › Supplementary Figure S1.pdf]

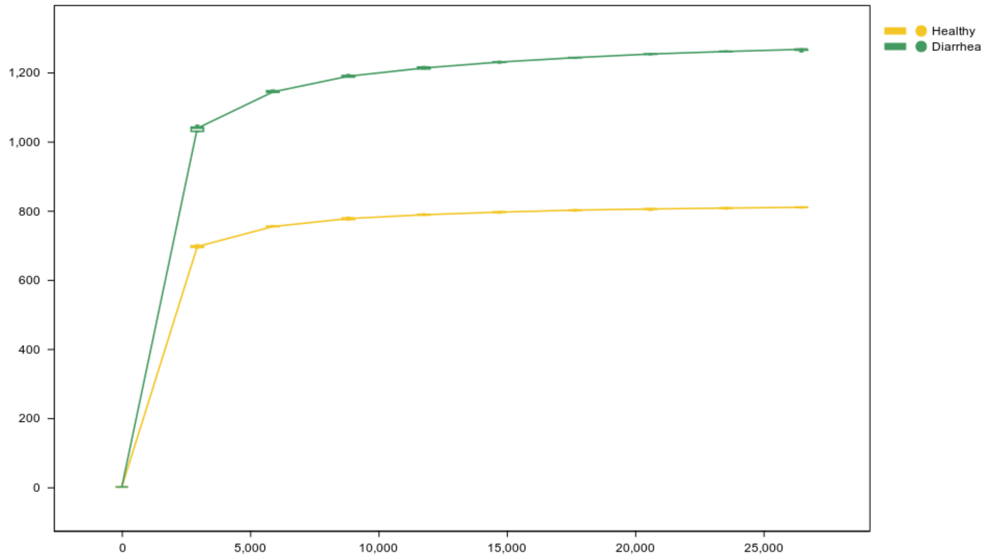

Supplementary Figure S1. Rarefaction curves of amplicon sequence variants (ASVs) for healthy and diarrhea groups. The x-axis represents the sequencing depth (number of reads per sample), and the y-axis shows the observed number of ASVs. The yellow line indicates healthy samples, while the green line represents diarrhea samples. The curves of both groups gradually plateaued at higher sequencing depths, indicating that the sequencing depth was sufficient to recover most microbial taxa in the samples.
